# Genome-Wide Identification of NAC Family Genes and Their Expression Analyses in Response to Osmotic Stress in *Cannabis sativa* L.

**DOI:** 10.3390/ijms25179466

**Published:** 2024-08-30

**Authors:** Qi Li, Hanxue Zhang, Yulei Yang, Kailei Tang, Yang Yang, Wenjing Ouyang, Guanghui Du

**Affiliations:** School of Agriculture, Yunnan University, Kunming 650500, China; liqi1@stu.ynu.edu.cn (Q.L.); zhanghanxue@mail.ynu.edu.cn (H.Z.); yylei@mail.ynu.edu.cn (Y.Y.); kailei.tang@ynu.edu.cn (K.T.); yjy@ynu.edu.cn (Y.Y.); wenjing.ouyang@ynu.edu.cn (W.O.)

**Keywords:** NAC transcription factor, *Cannabis sativa* L., osmotic stress, expression pattern

## Abstract

NAC (NAM, ATAF1/2, and CUC2) transcription factors are unique and essential for plant growth and development. Although the NAC gene family has been identified in a wide variety of plants, its chromosomal location and function in *Cannabis sativa* are still unknown. In this study, a total of 69 putative CsNACs were obtained, and chromosomal location analysis indicated that the CsNAC genes mapped unevenly to 10 chromosomes. Phylogenetic analyses showed that the 69 CsNACs could be divided into six subfamilies. Additionally, the CsNAC genes in group IV-a are specific to *Cannabis sativa* and contain a relatively large number of exons. Promoter analysis revealed that most CsNAC promoters contained cis-elements related to plant hormones, the light response, and abiotic stress. Furthermore, transcriptome expression profiling revealed that 24 CsNAC genes in two *Cannabis sativa* cultivars (YM1 and YM7) were significantly differentially expressed under osmotic stress, and these 12 genes presented differential expression patterns across different cultivars according to quantitative real-time PCR (RT–qPCR) analysis. Among these, the genes homologous to the *CsNAC18*, *CsNAC24*, and *CsNAC61* genes have been proven to be involved in the response to abiotic stress and might be candidate genes for further exploration to determine their functions. The present study provides a comprehensive insight into the sequence characteristics, structural properties, evolutionary relationships, and expression patterns of NAC family genes under osmotic stress in *Cannabis sativa* and provides a basis for further functional characterization of CsNAC genes under osmotic stress to improve agricultural traits in *Cannabis sativa*.

## 1. Introduction

*Cannabis sativa* L. is an annual herb of the Cannabaceae family whose fibers can be used for the textile industry, paper production, and construction and whose seeds can be used in food [1]. Research has revealed that the inflorescences and leaves of *Cannabis sativa* are rich in cannabidiol (CBD), which has anti-epileptic, anti-anxiety, anti-inflammatory, and other medicinal values [2]. The versatility of this crop has attracted great attention from the public and from researchers in the development of the *Cannabis sativa* industry. Owing to the shortage of cultivated land resources, most *Cannabis sativa* plants are planted on saline–alkali lands, hillside lands, and winter fallow lands [3], making them susceptible to osmotic stress (drought, salt, low temperature, etc.), which significantly influences growth, especially the germination of *Cannabis sativa* seeds. To mitigate these effects caused by adverse environmental factors, it is necessary to explore and identify genetic resources related to osmotic stress resistance in *Cannabis sativa*.

Gene expression is largely regulated by specific transcription factors (TFs) that control the rate of transcription of genetic information from DNA to mRNA by binding to a specific DNA sequence. Sessile plants cope with a variety of abiotic and biotic stresses by means of a strong regulatory mechanism that is modulated through many TFs. Well-studied TFs in plants include MIKC, C2H2, WRKY, bZIP, MYB, SBP, HB, AP2/EREBP, and NAC [4]. Among these plant gene families, the NAC gene family is one of the largest and most characteristic [5,6].

The name of the NAC gene family was derived from the initial names of the NAM (no apical meristem), AF1/2, and CUC2 (cup-shaped cotyledon) transcription factors, which contain highly conserved domains. The NAC domain is composed of an N-terminal region of nearly 150 amino acid residues in length and consisting of five (A–E) subdomains and an alterable C-terminal domain, which is predicted to bind to DNA as a transcriptional activator or repressor and to confer the functional diversity of NAC proteins [7,8].

Many studies have shown that the response to various abiotic stresses, such as heat stress, low-temperature stress, drought stress, and saline–alkali stress, is directly or indirectly regulated by NAC TFs [9,10,11,12,13]. The overexpression of *TaSNAC4-3A* in wheat has been reported to stimulate germination and root growth when it is exposed to salt and osmotic stresses [14]. Ma et al. reported that *TaNAC5D-2* is a positive regulator of drought tolerance in wheat and controls water loss under drought conditions through abscisic acid (ABA)-mediated stomatal closure [15]. *ZmSNAC13* and *ZmNAC071* in maize have been demonstrated to increase the effective photosynthesis rate and cell membrane stability under drought stress; additionally, they increase the sensitivity of transgenic *Arabidopsis thaliana* plants to ABA and osmotic stress [16,17]. The overexpression of *OsNAC2* in rice results in lower resistance to high salt and drought conditions, contrary to the effect of the RNAi lines of *OsNAC2* [18]. Jian et al. reported that the overexpression of *SlNAC6* greatly increased the proline content and antioxidant enzyme activity so that it enhanced the tolerance of tomatoes to drought stress [19]. In *Rosa chinensis*, *RcNAC27* was associated with the response to drought, low temperature, salt, and ABA treatments. In addition, the overexpression of *RcNAC72* in *Arabidopsis thaliana* increased the sensitivity to ABA and tolerance to drought stress [20]. In *Cucurbita moschata*, *CmNAC*1 is involved in ABA signaling pathways, and the ectopic expression of *CmNAC1* in *Arabidopsis thaliana* led to ABA hypersensitivity and increased tolerance to salinity, drought, and cold stresses [21]. The above studies have demonstrated that the NAC genes play important roles in plant responses to osmotic stresses such as drought and salt-alkali stress.

However, studies on the NAC genes have focused mainly on the model plant *Arabidopsis thaliana*, and there has been no systemic characterization of the NAC genes in *Cannabis sativa*. The number of NAC family members in *Cannabis sativa*, their related functions under osmotic stress, and their mode of action in different *Cannabis sativa* accessions have remained elusive. Therefore, in this study, multiple bioinformatics methods were used to identify the *Cannabis sativa* NAC gene family from the published genome of CBDRx female plants and comprehensive analyses, including gene structure, conserved motif, chromosomal location, and phylogenetic analyses, of the putative CsNACs were performed. The expression patterns of 12 potential stress-responsive NAC genes in two *Cannabis sativa* accessions during seed germination under osmotic stress were subsequently detected using qRT–PCR. The results provide a biological reference for future studies on the function of NAC genes and lay the foundation for the breeding of resistant varieties of *Cannabis sativa*.

## 2. Results

### 2.1. Genome-Wide Identification and Analysis of NAC Genes in Cannabis sativa

**Figure 1:**
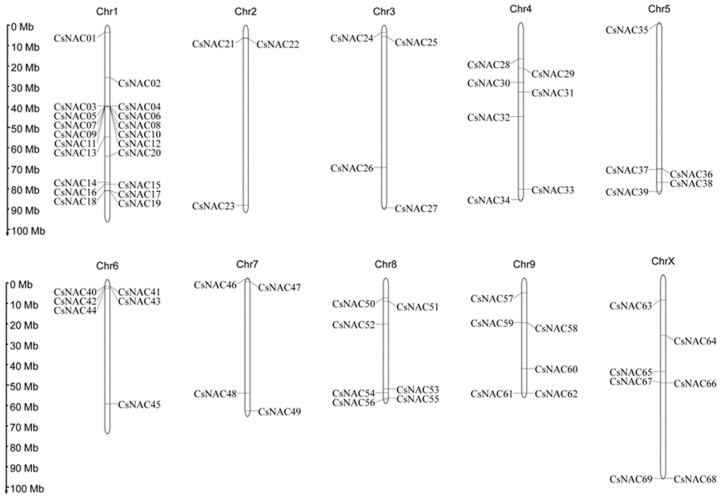
Physical mapping of CsNAC genes in the *Cannabis sativa* genome. The ten *Cannabis sativa* chromosomes are numbered from Chr1 to ChrX. CsNAC genes are numbered consecutively on the basis of their position on the chromosomes (CsNAC01-CsNAC69). The scale bar on the left shows the chromosome length in megabases (Mb).

With respect to the *Arabidopsis thaliana* NAC family protein sequence, 69 CsNACs were detected with HMM. On the basis of chromosomal location, the CsNACs were named *CsNAC1-CsNAC69* (Figure 1). Sixty-nine NAC-encoding genes were distributed unevenly on chromosomes 1 to 10 in *Cannabis sativa*. Chromosome 1 contained the greatest number of NAC genes (29.98%), followed by chromosomes 4, 8, and X (10.14%). In contrast, chromosome 3 contained only 4.34% of the NAC genes.

The sequence length of the CsNAC protein ranged from 136 aa to 860 aa, with the shortest sequence in CsNAC68 and the longest in CsNAC37, and with PI values ranging from 4.45 to 9.87, with the lowest value in CsNAC57, followed by CsNAC37, and the highest in CsNAC23. Furthermore, the MW ranged from 16.06 to 96.02 kDa, with a minimum of CsNAC4 and a maximum of CsNAC37 (Table 1).

**Figure 2:**
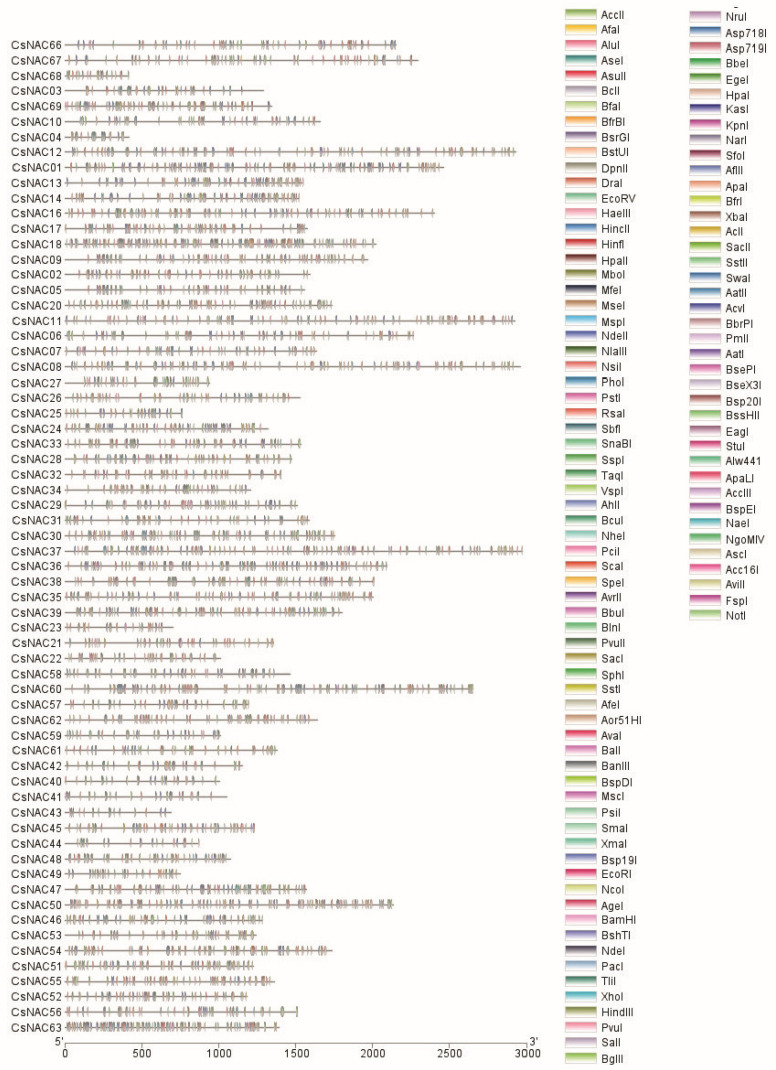
Restriction enzyme analysis patterns of CsNAC genes.

Furthermore, we conducted restriction endonuclease digestion patterns analysis, and a total of 26 sites for common restriction endonucleases were identified (Figure 2). The result showed that 69 CsNAC genes had distinguishable patterns, indicating that there was no redundancy among the 69 predicted NAC genes.

**Figure 3:**
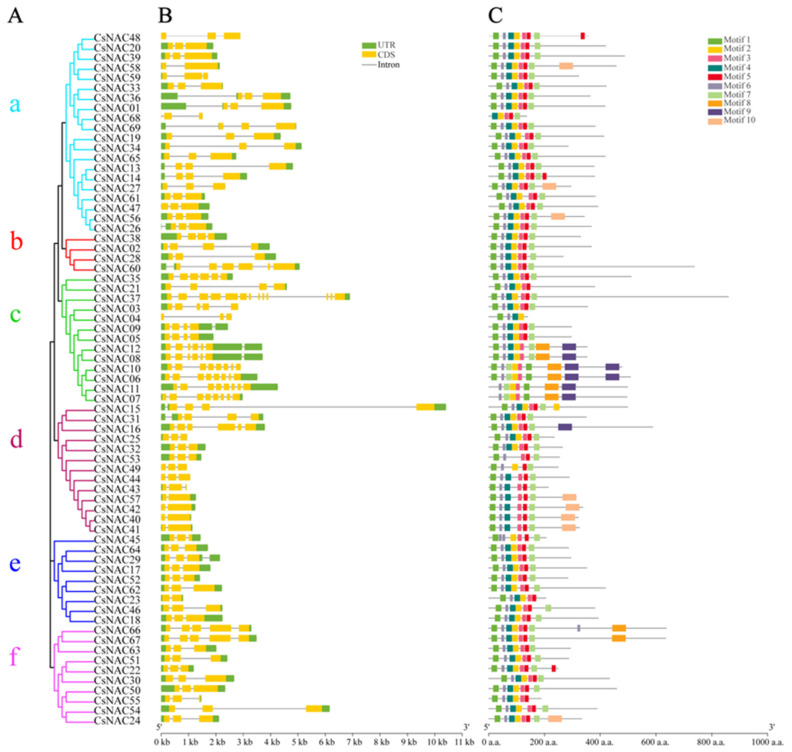
The phylogenetic tree (**A**) and the conservation motifs (**C**) of the CsNAC genes. UTRs, exons, and introns are represented by green boxes, yellow boxes, and black lines, respectively (**B**).

**Figure 4:**
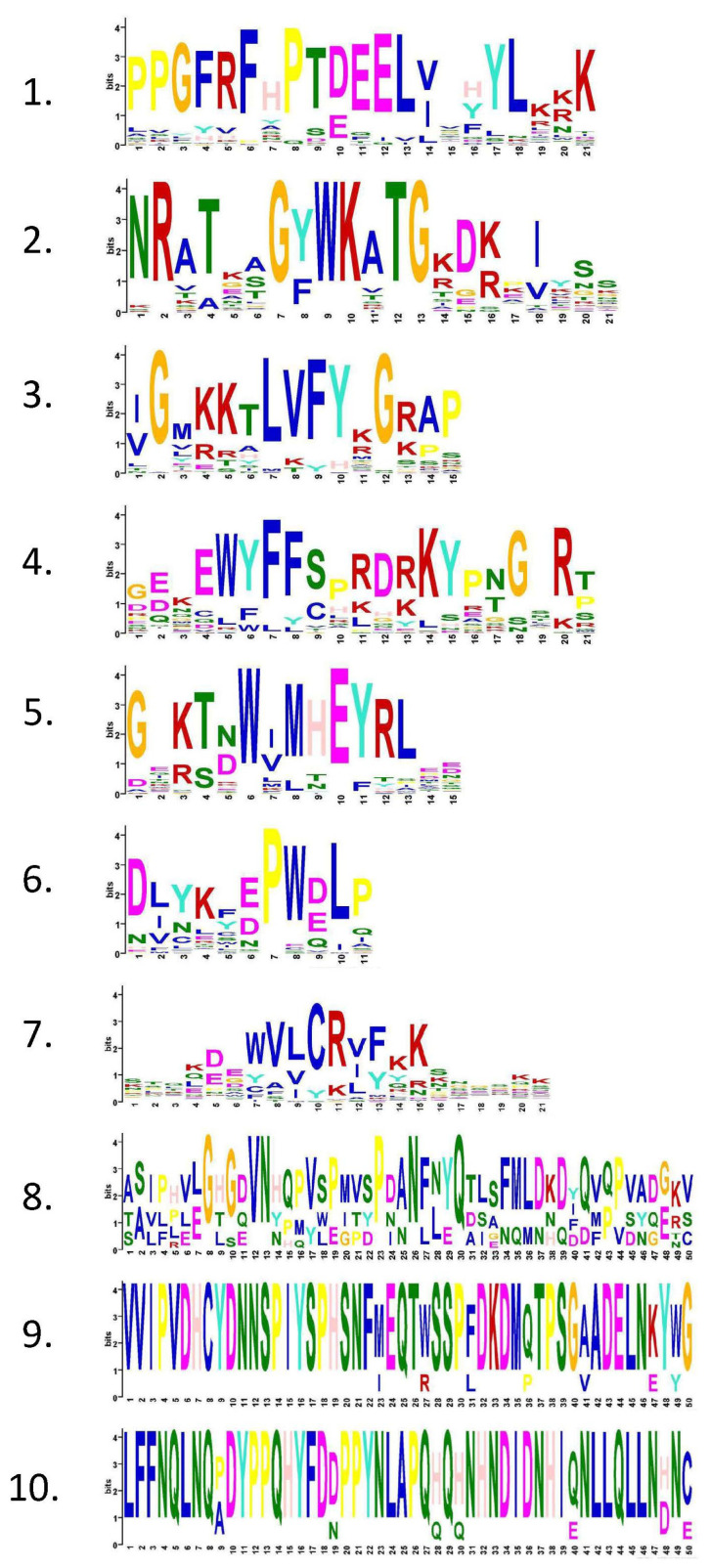
Conserved motif of CsNAC proteins.

To further understand the conservation and diversification of the 69 identified CsNAC proteins, the motif structures were predicted via the MEME program. The results revealed that 10 conserved motifs were distributed among various gene members (Figure 3C). The 10 different motifs identified in CsNAC were named motifs 1–10 (Figure 4). Among them, motifs 1, 3, 7, and 6 are present in all members of CsNAC genes. In addition, motifs 9 and 10 are only present in a small number of CsNAC genes. To further understand the structure of CsNAC genes, we analyzed their intron/exon composition (Figure 3B). The number of introns ranged from 1 to 12, and the number of exons ranged from 2 to 13, with CsNAC37 containing the largest number of introns and exons.

We predicted that approximately 97% of the CsNAC genes would be located in the nucleus, while a location in the endoplasmic reticulum (*CsNAC66* and *CsNAC67*) was predicted for the other genes (Table S1).

### 2.2. Identification Duplicated CsNAC Genes

**Figure 5:**
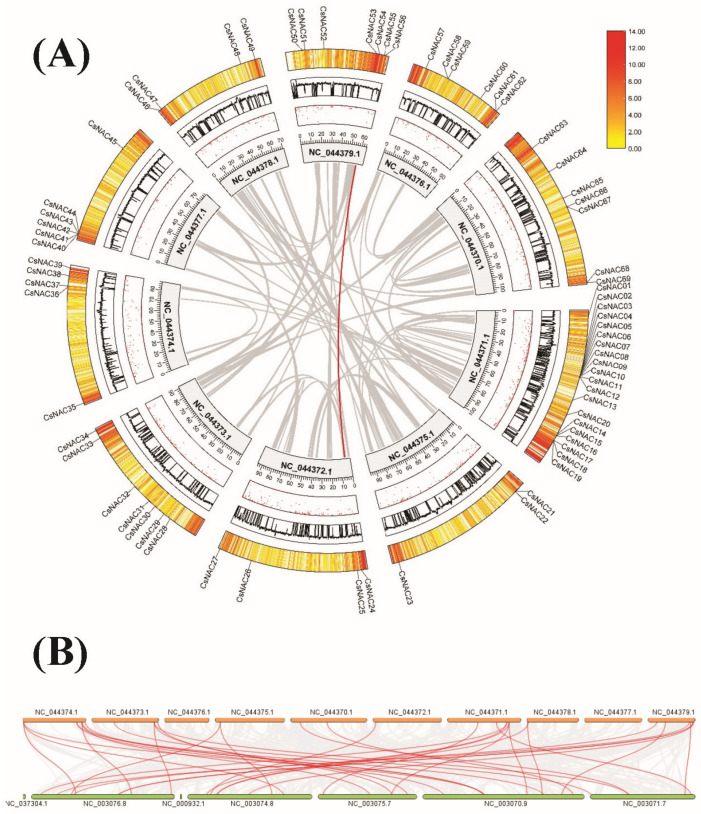
Interchromosomal relationships of CsNAC genes (**A**). Each plate represents *Cannabis sativa* chromosomes, GC content, density, and clustering heatmaps from the inside to the outside. Synteny analyses between *Cannabis sativa* and *Arabidopsis thaliana* (**B**). The gray lines in the background indicate collinear blocks within *Cannabis sativa* and *Arabidopsis thaliana*.

The potential mechanisms involved in the evolution of the NAC gene family in *Cannabis sativa* were further explicated by analyzing gene collinearity with the MCScanX tool of TBtools software v2.119. Only CsNAC56 and CsNAC25 were found to have segmental duplications (Figure 5A). These findings suggested that segmental duplications assisted in the expression of CsNAC genes in the *Cannabis sativa* genome and expanded the quantity of NAC genes and chromosome 10, primarily attributable to evolution.

To further analyze gene replication, the evolutionary relationship of NACs between *Arabidopsis thaliana* and *Cannabis sativa* was analyzed (Figure 5B), finding that of the 69 CsNAC genes, 22 had 34 pairs with collinearity with *Arabidopsis thaliana*. Half of the pairs were single pairs, while the other half had two or three pairs of NAC collinearities between *Arabidopsis thaliana* and *Cannabis sativa*.

### 2.3. Phylogeny of CsNAC Genes

**Figure 6:**
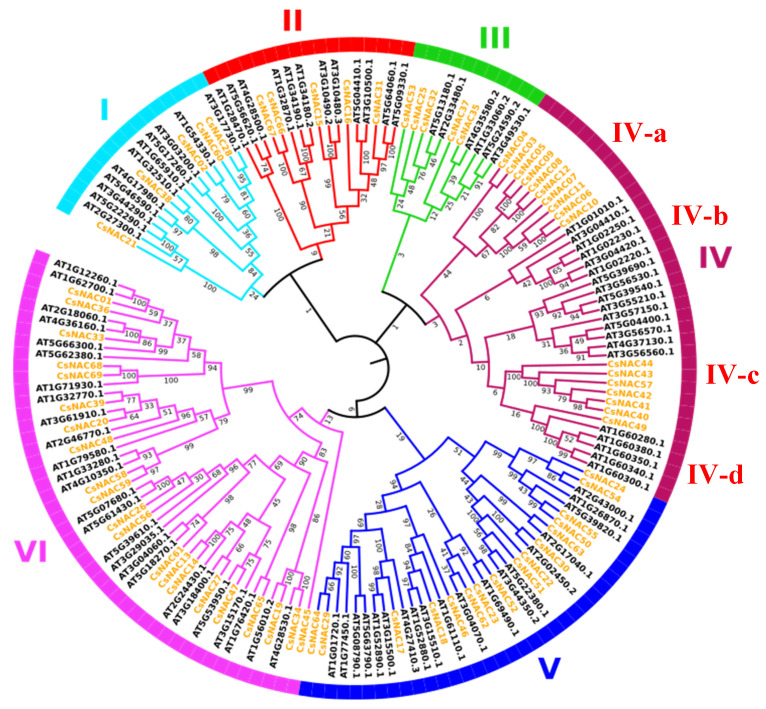
Evolutionary analysis of Cannabis sativa NAC genes. Each NAC subfamily is indicated with a specific color.

To better analyze the phylogenetic organization of the *Cannabis sativa* NAC family, the predicted protein sequences were used to generate a phylogenetic tree, dividing the CsNAC genes into six major groups (I–VI) according to the classification of AtNAC. Among the 69 CsNACs, group VI accounted for the most proteins (47), followed by group IV (37). Furthermore, group IV includes 4 subgroups: IV-a is a subgroup unique to *Cannabis sativa*, and IV-b and IV-c are subgroups unique to *Arabidopsis thaliana* (Figure 6). Furthermore, CsNAC genes in the same group contained similar numbers of exons and introns, among which subgroup b had the larger number (Figure 3B). Additionally, some of the motifs were ubiquitous in all CsNAC genes. Some CsNAC genes in subgroup c specifically contained motif 9 and were clustered separately in subgroup IV-a in the phylogenetic tree (Figure 3C).

### 2.4. Cis-Element Analysis of the Promoter Regions of the CsNAC Genes

**Figure 7:**
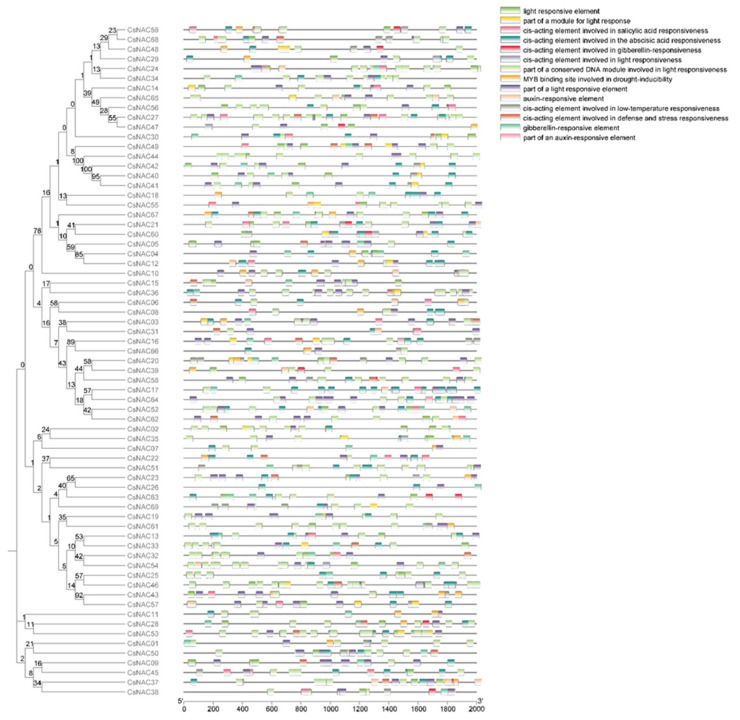
Putative cis-acting regulatory elements in the promoters of CsNAC genes. The number on nodes is the bootstrap value of each node.

The upstream 2000 bp sequences of all 69 CsNACs were retrieved, and the PlantCARE tool was used to predict their cis-acting features. A number of cis-acting elements were identified with different roles, such as hormone responsiveness elements, MYB binding domains, low-temperature responsiveness elements, defense and stress-related factors, and light responsiveness elements (Figure 7). Interestingly, nearly all the promoters of CsNAC genes contained multiple cis-acting elements related to light response. Additionally, a large number of salicylic acid-related (3.55%), gibberellin-related (5.58%), abscisic acid-related (18.33%), and auxin-related (2.33%) cis-acting elements are found in the CsNACs promoters (Table S2).

### 2.5. Interaction Analysis of the CsNAC Proteins

**Figure 8:**
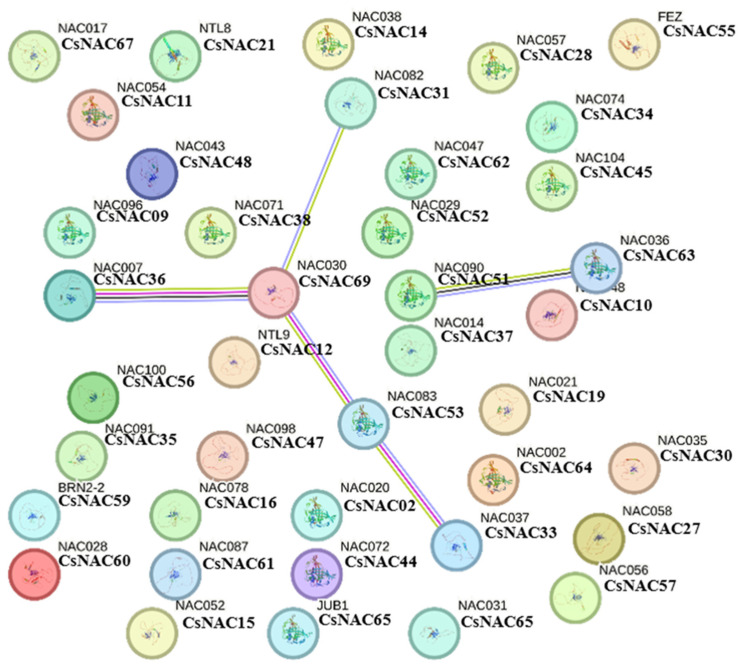
Protein–protein interactions of CsNACs visualized using the STRING 12.0 online tool with *Arabidopsis thaliana* as the reference genome.

With the STRING12.0 online tool, only CsNAC31, CsNAC69, CsNAC36, CsNAC53, CsNAC33, CsNAC51, and CsNAC63 strongly interacted with each other (Figure 8). This suggests that the PPI network of CsNACs might mediate signaling and process any biological and molecular functions through mutual interactions.

### 2.6. Transcriptome Sequencing of Cannabis sativa in Response to Osmotic Stress

To understand the transcription level of CsNAC in response to osmotic stress, transcriptome sequencing was performed on YM1 and YM7 seedlings that had germinated for 7 days under normal conditions and osmotic stress conditions. On average, each sample generated approximately 6.92 Gb of data, and the Q30 base percentage for each sample was not less than 95.23%. The reads of the sample were compared with the reference genome (GCF_900626175.2), and the comparison efficiency was between 86.68% and 90.69% (Table 2). After the alignment analysis was completed, StringTie was used to assemble and quantify the reads on the alignment and calculate the expression levels of genes in different samples. This showed that the CsNAC genes in YM1 and YM7 are differentially expressed under osmotic stress. Among the 69 CsNAC genes, the expression levels of 29 were obviously different in YM1 and 27 in YM7. Furthermore, 24 genes were differentially expressed in both YM1 and YM7 (Table 3). Other specific information on differentially expressed genes can be found in Tables S3 and S4.

### 2.7. Expression Analysis of CsNAC Genes in Response to Osmotic Stress

**Figure 9:**
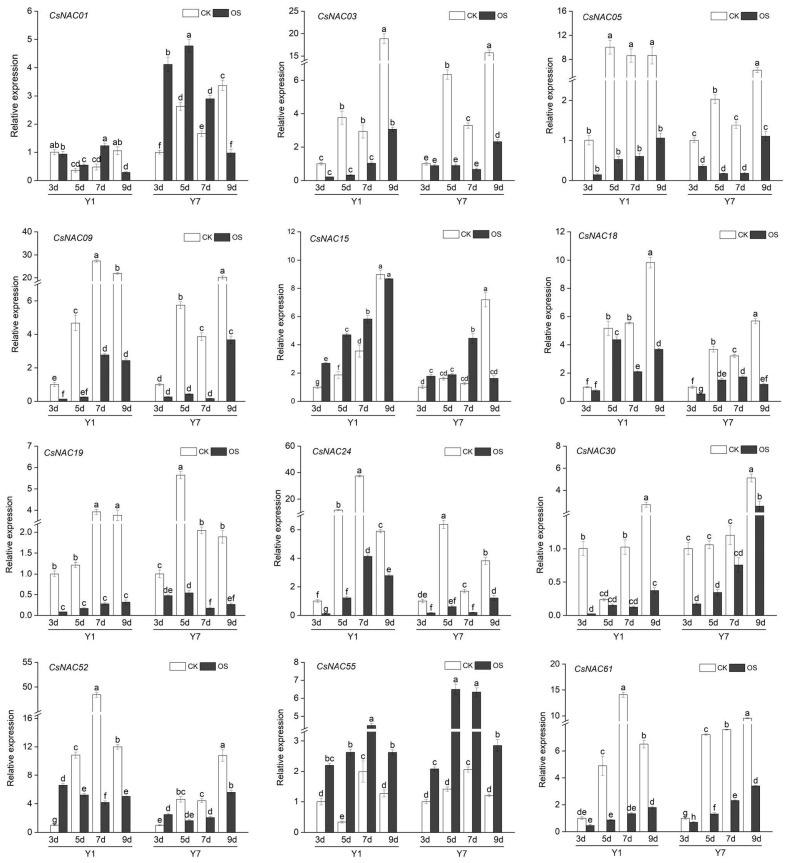
Relative expression of CsNAC genes in two different *Cannabis sativa* cultivars (Y1 is YM1 and Y7 is YM7) under normal germination treatment (CK) and osmotic stress treatment (OS). Seedlings were sampled at 3, 5, 7, and 9 days after germination. The different letters indicate significant differences at different times, according to Duncan’s multiple range test (*p* < 0.05).

To better determine the expression patterns of these genes, 12 CsNAC genes with significant expression differences in the transcriptomes of YM1 and YM7 under osmotic stress were selected for analysis by means of qRT-PCR (Figure 9). The results revealed relatively higher expression levels of *CsNAC01*, *CsNAC55*, and *CsNAC15* in YM1 under osmotic stress compared with normal conditions. The relative expression level of *CsNAC01* increased on the 7th day of seed germination, on the 5th and 7th days of germination for *CsNAC55*, and on the 3rd to 9th days of seed germination for *CsNAC15*. Additionally, relatively greater expression was detected in *CsNAC01*, *CsNAC15*, *CsNAC52*, and *CsNAC55* of YM7 under osmotic stress than under normal conditions. The relative expression level of *CsNAC52* increased on the 3rd day of germination, from the 3rd to the 7th day of germination for *CsNAC15* and *CsNAC55*, and from the 3rd to the 9th day of germination for *CsNAC55*. *CsNAC15* and *CsNAC61* in both *Cannabis sativa* accessions were induced by osmotic stress, and their relative expression levels gradually increased with germination time. However, the relative expression level of *CsNAC55* first increased and then decreased with increasing germination time, but *CsNAC52* first decreased but then increased with increasing germination time.

## 3. Discussion

### 3.1. Identification and Evolutionary Analysis of CsNAC Gene

NAC-type proteins, among the largest plant transcription factor family members, play important roles in many aspects of plant development processes, including the stress response, signaling pathways, and plant defenses. However, studies related to NAC genes in *Cannabis sativa* have not yet been reported. Therefore, we performed a genome-wide analysis of NAC transcription factors in the female plants of *Cannabis sativa* CBDRx-18 and explored the potential functions of homologous genes in YM1 and YM7 in coping with osmotic stress. Sixty-nine members of the CsNAC gene family were identified, significantly fewer than those of *Arabidopsis thaliana* (96) [22,23,24], rice (151) [25], soybean (151) [26], and maize (148) [27]. However, this is relatively more numerous than in some other crops, such as oilseed rape, with 60 [28]. These results suggest that the numbers of NAC genes do not match the genome size of the species, indicating that the NAC genes were stable during the process of evolution in different species. Additionally, genome duplication events occurred during the process of plant evolution, and the major duplication patterns were tandem and segmental duplication [29,30,31,32]. Although *CsNAC56* and *CsNAC25* were found to be segmental duplications, the main factors that drove the expansion of the CsNAC genes might not have been segmental duplications due to fewer duplication events. The analysis of gene structure revealed that the number of introns present in the CsNAC genes varied from 1 to 12, greater than that reported in soybean and cotton, in which the number of introns varied from 1 to 7 and 0 to 9, respectively [33,34]. These results suggest that the gene structure of the CsNAC genes is more diverse than that of the NAC genes in soybean and cotton. Furthermore, the exon–intron structure was similar in most of the CsNAC members that were present in the same group. In each group, close evolutionary relationships were supported by the conserved intron numbers [35]. A previous study reported similar results. The analysis of cis-elements in the promoter regions allowed the prediction of potential mechanisms of CsNAC gene regulation. The promoters of the CsNACs included defense and stress response elements (low-temperature and drought response elements), growth- and development-related elements (light response and auxin response elements), and hormone response elements (gibberellin, salicylic acid, and abscisic response elements), suggesting that the CsNAC genes are involved in the growth and development of *Cannabis sativa* and the process of coping with abiotic stress.

Phylogenetic analysis of the NAC gene family in *Cannabis sativa* and *Arabidopsis thaliana* showed that the CsNAC genes with similar motifs tend to cluster into one subgroup, and the differences were observed only in different subgroups, which might indicate functional similarity among gene members in the same subgroup [36]. For example, some genes, including *ATAF1* (At1g01720), *ANAC019* (At1g52890), and *RD26/ANAC072* (At4g27410), which belong to Group V, can increase plant resistance to abiotic stresses such as drought and high temperature [37,38,39,40]. It could be speculated that those CsNAC genes of the same subgroup may also be involved in the response to stress. Additionally, among those in group IV-a, 10 member proteins were clustered in a single branch, and all were located on chromosome 1, implying that tandem duplication contributed to the expansion of the NAC genes. Furthermore, there were all CsNAC genes in group IV-a, suggesting that this group might have been unique to *Cannabis sativa* during its evolution. Most genes in this group had more introns and CDSs, and most had motifs 8 and 9, indicating that these genes might have more splicing patterns and might lead to diverse gene functionality.

Network interaction relationship analysis of 69 CsNAC proteins showed that 7 genes formed interaction proteins, but 62 genes could not form interaction relationships. This indicates that these proteins have important roles. Significantly, CsNAC69, which is located in the center of the interaction network, was homologous to NAC30 in maize; *ZmNAC30* was found to be involved in stress responses and/or root growth and development [41]. In the future, it is worth further studying and verifying the role of this gene and NAC proteins interaction network in *Cannabis sativa*.

### 3.2. The Role of the CsNAC Genes in the Cannabis sativa Seed Germination Process under Osmotic Stress

According to the qRT-PCR results for the 12 CsNAC genes, compared with those of the CK, the relative expression of only 4–5 genes was obviously upregulated during either the early or late stage of osmotic stress, whereas the other genes were downregulated, indicating that osmotic stress might have an impact on the suppression of their expression. Moreover, there were differences in the expression of each gene between YM1 and YM7, but both were downregulated in the two cultivars. These results suggest that the expression of the CsNAC genes might be related to the cultivar. Compared with *Arabidopsis thaliana*, we found that *CsNAC15* was homologous to *ANAC050* (AT3G10480) and *ANAC052* (AT3G10490), which participate in transcriptional repression and delayed flowering by binding to *JMJ14* (histone H3K4 demethylase) [42]. *CsNAC24* was homologous to *JUB1* (At2g43000). Wu et al. reported that *JUB1* overexpression in plants delayed *Arabidopsis thaliana* plant cell senescence and decreased intracellular H_2_O_2_ levels, increasing tolerance to abiotic stress, whereas, in *JUB1* knockdown plants, precocious senescence and decreased abiotic stress tolerance were observed [43]. Moreover, a previous study reported that *JUB1* increased tolerance to heat stress in *Arabidopsis thaliana* when it was overexpressed [44]. The overexpression of the *CsNAC30* homologous gene *LOV1* (AT2G02450) in switchgrass (*Panicum virgatum*) altered the lignin content and monolignol composition of the cell wall and delayed flowering [45]. *CsNAC52* is homologous to *NAP* (AT1G69490), which plays a role in positively regulating age-dependent and dark-induced leaf senescence through the GA pathway [46]. *CsNAC18* was homologous to *NAM* (AT1G52880) and *NAC25* (At1g61110). *NAM* in upland cotton was negatively regulated by salt stress, drought stress, H_2_O_2_ stress, IAA treatment, and ethylene treatment but positively regulated by ABA and MeJA treatment. However, its heterologous overexpression results in premature leaf senescence and delayed root system development in *Arabidopsis thaliana* [47]. *NAC25* was identified as a regulator of endosperm cell expansion controlling the seed-to-seedling transition [48]. *CsNAC19* and *CsNAC61* share high identity with *NAC1* (At1g56010) and *ORS1* (AT3G29035), respectively. Previous studies have shown that *NAC1* maintains root meristem size and root growth by directly repressing the transcription of *E2Fa* in *Arabidopsis thaliana* [49] and that the overexpression of *ORS1* accelerates senescence in *Arabidopsis thaliana*, whereas its inhibition delays senescence [50]. *CsNAC01* was homologous to *VND4* (AT1G12260) and *VND5* (AT1G62700), which serve as transcriptional regulators that participate in secondary wall biosynthesis [51].

Genes in the same subgroup of a phylogenetic tree often have the same functional features. In this study, *CsNAC18*, *CsNAC24*, and *CsNAC61* might participate in the response to abiotic stress because their homologous genes, which clustered in the same subgroup, have been previously identified as stress-response genes. The identification of several CsNAC genes in the present study provided clues for the selection of candidate genes for further studies.

## 4. Materials and Methods

### 4.1. Identification of NACs in the Cannabis sativa Genome

The draft genome of *Cannabis sativa* was downloaded from NCBI (GCF_900626175.2). For the identification of orthologs of NACs in *Cannabis sativa*, the NAC protein sequence of *Arabidopsis thaliana* was obtained from TAIR (http://www.arabidopsis.org/, accessed on 22 April 2022). BioEdit, was used to conduct BLAST analysis, in which NAC transcription factors exhibiting significant homology were identified, and redundancies were removed. All the candidate genes were subsequently verified using the hidden Markov model (PF02365) of the NAC gene domain by using the Pfam tool (http://pfam.xfam.org/search, accessed on 18 July 2022). ExPASy (http://cn.ExPASy.org/tools, accessed on 17 July 2022) was utilized to predict key physicochemical properties such as the isoelectric point (PI), molecular weight (MW), and other pertinent characteristics of the CsNAC protein. The restriction endonuclease digestion patterns of the 69 CsNACs were obtained from NovoPro (https://www.novopro.cn/tools/rest_map.html, accessed on 20 August 2024) and were visualized using TBtools.

### 4.2. Phylogenetic Analysis of CsNAC

The AtNAC protein sequences in *Arabidopsis thaliana* and the identified CsNAC in *Cannabis sativa* were aligned using MUSCLE. On the basis of the alignment results, MEGA X software was used to generate a phylogenetic tree with the Jones–Taylor–Thornton (JTT) model (bootstrap = 1000).

### 4.3. Chromosomal Location, Gene Structure and Motif Analysis of CsNACs

The chromosomal location information for the CsNAC family genes was obtained using the MG2C tool (http://mg2c.iask.in/mg2c_v2.1/, accessed on 19 July 2022). Motif analysis was performed with the MEME program (functional domains = 10). All figures were generated using the TBtools software v2.119.

### 4.4. Sub-Cellular Location of CsNAC

We predicted the sub-cellular localization of CsNAC genes by submitting their protein sequences to Cell-PLoc 2.0 (https://www.sohu.com/a/149196044_278730, accessed on 21 May 2024).

### 4.5. Cis-Regulatory Features in the Upstream Promoter Regions of CsNACs

To analyze the cis-acting features in the upstream promoter regions of the 69 putative CsNACs, a 2000 bp DNA sequence upstream of the CsNACs was retrieved from the genome data, and possible cis-acting elements were predicted using PlantCARE (http://bioin-formatics.psb.ugent.be/webtools/PlantCARE/html, accessed on 5 April 2023). TBtools software v2.119was used to generate graphical figures.

### 4.6. Protein-Protein Interaction Studies of CsNAC

To analyze the protein–protein interactions (PPIs), the STRING 12.0 database (http://string-db.org/, accessed on 5 April 2023) was employed, utilizing the default parameters and *Arabidopsis thaliana* as the reference organism.

### 4.7. RNA-Seq Analysis

#### 4.7.1. Plant Materials

Two *Cannabis sativa* cultivars, ‘Yunma 1, YM1’ and ‘Yunma 7, YM7’, were used in this study. Two cultivars had with different tolerance to osmotic stress, and YM7 had higher resistance. Both cultivars were obtained from Yunnan Industrial Hemp Co., Ltd., Kunming, China. After sterilization with 70% alcohol, rinsing with distilled water, and drying, 30 seeds containing particles of consistent sizes were selected and placed evenly in 10 cm petri dishes that were sterilized and lined with filter paper. A petri dish supplemented with 8 mL of distilled water was used for the normal germination treatment, and 8 mL of 20% PEG-6000 was added for the osmotic stress treatment. Three replicates were performed for each treatment. After the seeds were grown in the dark for 3 d, they were transferred to a controlled plant growth chamber with a light duration of 12 h (20 °C) and a 12 h (25 °C) photoperiod for 4 d. Five germinated seedlings from each biological replicate were frozen in liquid nitrogen and stored at −80 °C.

#### 4.7.2. RNA-Seq and Bioinformatics Analysis

RNA was extracted from the stored frozen samples strictly following the manufacturer’s instructions for the RNAprep Pure Plant Plus Kit (TIANGEN BIOTECH Co., Ltd., Beijing, China), after which cDNA libraries were constructed and sequenced on the Illumina sequencing platform by Biomarker Co., Ltd. (Beijing, China). The clean reads were mapped to the reference genome (GCF_900626175.2) using HISAT2 [52]. After the alignment analysis was completed, StringTie was used to assemble the aligned reads [53]. The maximum flow algorithm fragments per kilobase of transcript per million fragments mapped (FPKM) [54] was used as an indicator to measure transcript or gene expression levels. The differentially expressed genes (DEGs) between samples were identified with DESeq2 [55], and a fold change ≥1.5 and FDR < 0.05 were considered the thresholds.

### 4.8. qRT-PCR Analysis of CsNACs under Osmotic Stress

The real-time quantitative PCR (qRT-PCR) mixture was prepared using the MonAmpTM SYBR^®^ Greeb qPCR Mix (Low Rox) Knit ((Monad Biotech Co., Ltd., Wuhan, China), and the reaction was performed on an Applied Biosystems QuantStudio 7 Flex real-time machine (Thermo Fisher Scientific Inc., Waltham, MA, USA) with three technical replicates for each biological replicate. The reaction system (20 µL) consisted of MonAmpTM SYBR^®^ Green qPCR Mix (10 µL), forward and reverse primers (0.4 µL), cDNA (1 µL), and ddH_2_O (8.2 µL). The reaction conditions were as follows: 95 °C for 30 s; 40 cycles of 10 s at 95 °C and 30 s at 60 °C; and 15 s at 95 °C for the melting curve analysis. The qPCR results were calculated using the relative delta delta Ct (2^–ΔΔCt^) method (*eIF4E* was used as a reference gene). The primers used for qPCR are listed in Table S5.

### 4.9. Statistical Analysis

All the data collected were organized, and tables were drawn using Excel 2016. The Duncan method was employed for post hoc comparisons with different significance levels denoted by *p* < 0.05. Graphical presentation was carried out using OriginPro 2021 (9.8.0.200) software.

## 5. Conclusions

A complete chromosomal-based analysis of CsNAC genes from the *Cannabis sativa* genome identified 69 putative candidate genes classifiable into six groups and distributed over all 10 chromosomes. Gene structure, protein analysis, and phylogenetic analysis revealed that the CsNAC family was conserved during their evolution. Additionally, the similar structures and motif arrangements of the CsNAC proteins within the subfamilies further supported the classification predicted by the phylogenetic tree. Moreover, gene expression analysis revealed that the putative CsNACs involved in osmotic stress were differentially expressed between the two *Cannabis sativa* cultivars, suggesting their role in environmental and cultivar interactions. *CsNAC18*, *CsNAC24*, and *CsNAC61* are similar to *NAM* (At1G52880), *NAC25* (At1g61110), *JUB1* (At2g43000), and *ORS1* (At3G29035), respectively, and might be candidate genes for further exploration of their functions in regulating the growth and development of *Cannabis sativa* under osmotic stress. This study provides a comprehensive, high-quality chromosome-based identification of the NAC gene family in *Cannabis sativa*, facilitating further exploration of the molecular utility of CsNAC genes.

## Figures and Tables

**Table 1:** Information on the NAC gene family members in *Cannabis sativa*.

Gene Symbol	Gene ID	Peptide Length	Chromosome Number	Isoelectric Point	Molecular Weight (KDa)
*CsNAC01*	LOC115704795	417	Chr1	6.15	48.27
*CsNAC02*	LOC115706643	368	Chr1	4.88	42.34
*CsNAC03*	LOC115704045	355	Chr1	5.13	40.05
*CsNAC04*	LOC115704046	139	Chr1	5.49	16.06
*CsNAC05*	LOC115707050	296	Chr1	5.47	34.45
*CsNAC06*	LOC115708284	508	Chr1	5.38	56.52
*CsNAC07*	LOC115708285	496	Chr1	5.26	55.96
*CsNAC08*	LOC115708331	353	Chr1	5.78	40.64
*CsNAC09*	LOC115706598	297	Chr1	5.30	34.47
*CsNAC10*	LOC115704050	478	Chr1	5.60	53.15
*CsNAC11*	LOC115708192	498	Chr1	5.15	56.17
*CsNAC12*	LOC115704732	353	Chr1	5.57	40.80
*CsNAC13*	LOC115704782	378	Chr1	8.84	42.51
*CsNAC14*	LOC115705946	380	Chr1	8.84	42.69
*CsNAC15*	LOC115706009	498	Chr1	6.02	55.09
*CsNAC16*	LOC115706004	588	Chr1	4.54	65.40
*CsNAC17*	LOC115706266	352	Chr1	8.50	39.49
*CsNAC18*	LOC115706270	393	Chr1	7.31	43.35
*CsNAC19*	LOC115706318	413	Chr1	7.23	46.34
*CsNAC20*	LOC115708111	419	Chr1	5.81	47.31
*CsNAC21*	LOC115718596	380	Chr2	6.68	42.94
*CsNAC22*	LOC115718718	248	Chr2	6.86	28.73
*CsNAC23*	LOC115718524	205	Chr2	9.87	23.60
*CsNAC24*	LOC115710269	333	Chr3	6.57	39.24
*CsNAC25*	LOC115710199	235	Chr3	9.06	27.15
*CsNAC26*	LOC115709772	368	Chr3	9.00	41.39
*CsNAC27*	LOC115708730	295	Chr3	7.18	34.27
*CsNAC28*	LOC115712266	267	Chr4	5.22	30.86
*CsNAC29*	LOC115713726	295	Chr4	6.08	33.99
*CsNAC30*	LOC115714610	433	Chr4	6.81	48.79
*CsNAC31*	LOC115713981	350	Chr4	4.76	39.01
*CsNAC32*	LOC115712846	265	Chr4	9.43	30.31
*CsNAC33*	LOC115712070	422	Chr4	6.31	49.01
*CsNAC34*	LOC115712883	285	Chr4	5.63	32.92
*CsNAC35*	LOC115716209	511	Chr5	4.98	58.08
*CsNAC36*	LOC115715739	364	Chr5	6.30	41.75
*CsNAC37*	LOC115715736	860	Chr5	4.50	96.02
*CsNAC38*	LOC115715828	329	Chr5	5.28	37.09
*CsNAC39*	LOC115717256	487	Chr5	6.35	55.32
*CsNAC40*	LOC115725272	322	Chr6	4.50	37.95
*CsNAC41*	LOC115725395	325	Chr6	4.51	38.22
*CsNAC42*	LOC115725039	338	Chr6	4.51	39.68
*CsNAC43*	LOC115694863	214	Chr6	5.27	25.28
*CsNAC44*	LOC115694873	289	Chr6	5.40	33.73
*CsNAC45*	LOC115725662	206	Chr6	5.03	23.81
*CsNAC46*	LOC115697858	381	Chr7	8.97	43.12
*CsNAC47*	LOC115697141	392	Chr7	7.20	43.33
*CsNAC48*	LOC115696687	359	Chr7	7.74	41.40
*CsNAC49*	LOC115696790	249	Chr7	8.70	29.41
*CsNAC50*	LOC115698713	459	Chr8	6.43	51.65
*CsNAC51*	LOC115698928	287	Chr8	7.09	32.60
*CsNAC52*	LOC115701358	284	Chr8	6.96	32.57
*CsNAC53*	LOC115698755	254	Chr8	9.53	29.21
*CsNAC54*	LOC115698787	389	Chr8	6.09	44.69
*CsNAC55*	LOC115700780	188	Chr8	9.24	21.72
*CsNAC56*	LOC115701489	343	Chr8	7.20	39.82
*CsNAC57*	LOC115721938	314	Chr9	4.45	36.72
*CsNAC58*	LOC115723181	457	Chr9	6.84	52.16
*CsNAC59*	LOC115723676	323	Chr9	9.61	37.64
*CsNAC60*	LOC115721797	738	Chr9	5.56	83.83
*CsNAC61*	LOC115724173	382	Chr9	6.20	43.78
*CsNAC62*	LOC115723252	419	Chr9	8.24	48.33
*CsNAC63*	LOC115703936	293	ChrX	6.92	33.77
*CsNAC64*	LOC115709817	287	ChrX	6.33	32.83
*CsNAC65*	LOC115711844	418	ChrX	7.21	46.82
*CsNAC66*	LOC115712310	637	ChrX	4.54	72.14
*CsNAC67*	LOC115712323	635	ChrX	4.66	71.94
*CsNAC68*	LOC115696969	136	ChrX	9.79	16.09
*CsNAC69*	LOC115702348	382	ChrX	6.41	44.06

**Table 2:** Basic transcriptome data of *Cannabis sativa* cultivars ‘YM1’ and ‘YM7’.

Samples	Clean Bases	% ≥ Q30	Mapped Reads
YM1-CK1	6,670,487,864	0.9571	39,785,426 (89.23%)
YM1-CK2	6,749,721,064	0.9554	40,343,097 (89.44%)
YM1-CK3	6,489,204,412	0.9552	39,105,425 (90.16%)
YM1-T1	7,085,565,094	0.9538	42,580,022 (89.92%)
YM1-T2	6,935,724,352	0.9574	41,715,006 (90.00%)
YM1-T3	6,887,479,172	0.9532	41,339,511 (89.81%)
YM7-CK1	6,902,265,136	0.9553	39,989,676 (86.68%)
YM7-CK2	7,571,531,100	0.9566	44,674,940 (88.27%)
YM7-CK3	6,326,231,956	0.9574	37,321,714 (88.28%)
YM7-T1	6,491,325,706	0.9523	39,158,112 (90.27%)
YM7-T2	7,173,656,642	0.9580	43,483,607 (90.69%)
YM7-T3	7,793,387,594	0.9557	46,930,775 (90.11%)

‘CK’ and ‘T’ represent the normal germination treatment and osmotic stress treatment, respectively.

**Table 3:** Differential expression of CsNAC genes in *Cannabis sativa* cultivars ‘YM1’ and ‘YM7’ under osmotic stress.

YM1				YM7			
Gene	Gene ID	Regulated	FDR Value	Gene	Gene ID	Regulated	FDR Value
*CsNAC54*	LOC115698787	down	7.38 × 10^−5^	*CsNAC54*	LOC115698787	down	4.80 × 10^−3^
*CsNAC55*	LOC115700780	up	2.50 × 10^−2^	*CsNAC55*	LOC115700780	up	1.66 × 10^−18^
*CsNAC52*	LOC115701358	down	3.25 × 10^−9^	*CsNAC52*	LOC115701358	down	7.67 × 10^−5^
*CsNAC63*	LOC115703936	down	2.81 × 10^−4^	*CsNAC63*	LOC115703936	down	5.77 × 10^−3^
*CsNAC03*	LOC115704045	down	8.64 × 10^−5^	*CsNAC03*	LOC115704045	down	1.26 × 10^−4^
*CsNAC01*	LOC115704795	up	4.27 × 10^−6^	*CsNAC01*	LOC115704795	up	3.22 × 10^−4^
*CsNAC16*	LOC115706004	down	3.60 × 10^−6^	*CsNAC16*	LOC115706004	down	7.28 × 10^−4^
*CsNAC15*	LOC115706009	up	9.94 × 10^−17^	*CsNAC15*	LOC115706009	up	5.19 × 10^−13^
*CsNAC17*	LOC115706266	down	4.25 × 10^−6^	*CsNAC17*	LOC115706266	down	1.30 × 10^−3^
*CsNAC18*	LOC115706270	down	5.47 × 10^−14^	*CsNAC18*	LOC115706270	down	1.40 × 10^−19^
*CsNAC19*	LOC115706318	down	2.48 × 10^−60^	*CsNAC19*	LOC115706318	down	1.16 × 10^−16^
*CsNAC09*	LOC115706598	down	5.82 × 10^−25^	*CsNAC09*	LOC115706598	down	5.34 × 10^−6^
*CsNAC05*	LOC115707050	down	2.65 × 10^−10^	*CsNAC05*	LOC115707050	down	4.76 × 10^−2^
*CsNAC26*	LOC115709772	down	4.70 × 10^−10^	*CsNAC26*	LOC115709772	down	1.60 × 10^−3^
*CsNAC24*	LOC115710269	down	1.03 × 10^−4^	*CsNAC24*	LOC115710269	down	2.20 × 10^−2^
*CsNAC66*	LOC115712310	down	1.00 × 10^−6^	*CsNAC66*	LOC115712310	down	6.81 × 10^−8^
*CsNAC67*	LOC115712323	down	3.57 × 10^−3^	*CsNAC67*	LOC115712323	down	1.43 × 10^−4^
*CsNAC32*	LOC115712846	down	4.22 × 10^−16^	*CsNAC32*	LOC115712846	down	1.90 × 10^−14^
*CsNAC34*	LOC115712883	down	1.50 × 10^−11^	*CsNAC34*	LOC115712883	down	2.59 × 10^−5^
*CsNAC29*	LOC115713726	down	4.08 × 10^−20^	*CsNAC29*	LOC115713726	down	1.67 × 10^−5^
*CsNAC30*	LOC115714610	down	8.91 × 10^−6^	*CsNAC30*	LOC115714610	down	6.62 × 10^−9^
*CsNAC62*	LOC115723252	down	1.93 × 10^−25^	*CsNAC62*	LOC115723252	down	5.94 × 10^−6^
*CsNAC61*	LOC115724173	down	3.45 × 10^−8^	*CsNAC61*	LOC115724173	down	5.00 × 10^−4^
*CsNAC45*	LOC115725662	down	1.67 × 10^−3^	*CsNAC45*	LOC115725662	down	8.44 × 10^−4^
*CsNAC46*	LOC115697858	up	1.40 × 10^−2^	*CsNAC13*	LOC115704782	down	5.05 × 10^−8^
*CsNAC51*	LOC115698928	up	4.59 × 10^−3^	*CsNAC02*	LOC115706643	up	3.50 × 10^−2^
*CsNAC37*	LOC115715736	up	2.08 × 10^−4^	*CsNAC20*	LOC115708111	down	1.53 × 10^−9^
*CsNAC38*	LOC115715828	down	1.37 × 10^−5^				
*CsNAC21*	LOC115718596	up	2.57 × 10^−6^				

## Data Availability

Genomic data were collected from the NCBI database (https://www.ncbi.nlm.nih.gov/, accessed on 1 April 2022). Cis-elements were obtained from the PlantCARE database (http://bioinformatics.psb.ugent.be/webtools/plantcare/html/, accessed on 5 April 2023). *Arabidopsis thaliana* sequence information was downloaded from TAIR (https://www.arabidopsis.org/, accessed on 22 April 2022). Transcriptome data has been uploaded to the SRA database (project ID: PRJNA1149188). All databases in this study are available to the public.

## Supplementary Materials

The following supporting information can be downloaded at: https://www.mdpi.com/article/10.3390/ijms25179466/s1.
